# Stimulus-triggered enhancement of chilling tolerance in zebrafish embryos

**DOI:** 10.1371/journal.pone.0171520

**Published:** 2017-02-06

**Authors:** Bernadett Faragó, Tímea Kollár, Katalin Szabó, Csilla Budai, Eszter Losonczi, Gergely Bernáth, Zsolt Csenki-Bakos, Béla Urbányi, Csaba Pribenszky, Ákos Horváth, Judit Cserepes

**Affiliations:** 1 Applied Cell Technology Ltd., Budapest, Hungary; 2 Szent István University, Department of Aquaculture, Gödöllő, Hungary; 3 University of Veterinary Science, Faculty of Animal Hygiene and Herdhealth and Veterinary Ethology, Budapest, Hungary; National University of Singapore, SINGAPORE

## Abstract

**Background:**

Cryopreservation of zebrafish embryos is still an unsolved problem despite market demand and massive efforts to preserve genetic variation among numerous existing lines. Chilled storage of embryos might be a step towards developing successful cryopreservation, but no methods to date have worked.

**Methods:**

In the present study, we applied a novel strategy to improve the chilling tolerance of zebrafish embryos by introducing a preconditioning hydrostatic pressure treatment to the embryos. In our experiments, 26-somites and Prim-5 stage zebrafish embryos were chilled at 0°C for 24 hours after preconditioning. Embryo survival rate, ability to reach maturation and fertilizing capacity were tested.

**Results:**

Our results indicate that applied preconditioning technology made it possible for the chilled embryos to develop normally until maturity, and to produce healthy offspring as normal, thus passing on their genetic material successfully. Treated embryos had a significantly higher survival and better developmental rate, moreover the treated group had a higher ratio of normal morphology during continued development. While all controls from chilled embryos died by 30 day-post-fertilization, the treated group reached maturity (~90–120 days) and were able to reproduce, resulting in offspring in expected quantity and quality.

**Conclusions:**

Based on our results, we conclude that the preconditioning technology represents a significant improvement in zebrafish embryo chilling tolerance, thus enabling a long-time survival. Furthermore, as embryonic development is arrested during chilled storage this technology also provides a solution to synchronize or delay the development.

## Introduction

Cryopreservation of gametes and embryos conserves biological resources. This technique has successfully been applied in various areas, including assisted human reproduction [1–3], livestock breeding [4] and preservation of various species [5,6]. However, cryopreservation of zebrafish embryos remains unsuccessful to date. Many obstacles prevent successful zebrafish embryo cryopreservation:, a) highly impermeable chorion, b) high chilling sensitivity and c) different water- and cryoprotectant permeability of various embryo compartments [7–9]. Several cryopreservation techniques have been tested. Slow freezing has failed as a method because intracellular ice formation was inevitable, regardless of cryoprotectants or the use of aquaporins inserted into embryo membranes [10]. Several studies tested vitrification of embryos from various fish species including zebrafish, however they resulted in either zero or very limited survival, moreover none reported successful continued development passing the larval stage [11–14].

Most studies focus on the high chilling sensitivity of fish embryos as one of the main obstacles for a successful cryopreservation protocol. Various methods were used to reduce fish embryo chilling injuries including embryonic dechorionation to facilitate cryoprotectant penetration into embryos [14], selection of advanced embryonic stages that are more likely to survive [7,15,16], using of cryoprotectants to increase chilling tolerance [17–21], or partial removal of the yolk from advanced-stage embryos [22], but none were successful. The major factors affecting survival of chilled embryos include duration and temperature of exposure [16].

Over recent decades, zebrafish have gained prominence as an important model organism across disciplines such as developmental biology, genetics, physiology, toxicology and environmental genomics [23]. Additionally, comparative genomics between zebrafish and humans has revealed a considerable amount of genetic homology. The high degree of similarity with the human genome has propelled zebrafish as an important model organism for human disease [24]. Consequently, the number of genetically modified zebrafish lines is rapidly growing. However, the preservation of the numerous genetic variants is still the major problem.

While research and development in cryopreservation mainly aims to modify or refine existing procedures, a recently published technique exists that puts the cells themselves into the focus. This procedure involved a mild, cell-specific stimulus by hydrostatic pressure treatment (PTAT: pressure triggered activation of tolerance, formerly referred to as HHP or HP treatment) to prepare cells for an upcoming stress factor (e.g., the ones associated with cryopreservation such as mechanic and osmotic stresses and the toxic effects of the cryoprotectants). As a consequence, cell competency is improved together with continued development, differentiation and performance [25]. PTAT-related studies have shown improvement in the cryotolerance of various cell types, e.g. mouse and bovine embryos [26–28]; porcine and bovine sperm [29]; porcine, bovine, mouse and human oocytes [30,31]; and umbilical cord blood [32]. In general, cells treated with PTAT perform better in terms of cryosurvival, speed of recovery, continued *in vitro* development, fertilizing ability, embryo quality (number of cells, ratio of necrotic, picnotic index), *in vitro* and *in vivo* developmental potential and live birth rate [30,33–35].

Recently, a possible molecular mechanism was uncovered regarding how PTAT treatment improves bovine embryo cryosurvival [26]. Gene ontology analysis indicated that proper PTAT treatment promotes embryo competence through down-regulation of genes involved in cell death and apoptosis and up-regulation of genes involved in RNA processing, cellular growth and proliferation. Overall, PTAT treatment enhanced the competence of blastocysts through modest transcriptional changes.

The objective of our study was to investigate whether PTAT treatment improves the chilling tolerance of zebrafish embryos in terms of post-hatch survival. We further hypothesized that PTAT-treated chilled embryos can develop into adult fish and that their reproductive performance would be physiological.

## Materials and methods

### Animals and housing conditions

Wild-type zebrafish (*Danio rerio*) embryos of AB strain were used in all experiments. Zebrafish was obtained from the breeding unit of Department of Aquaculture, Szent István University, Gödöllő, Hungary. Parents (egg/sperm donors) were housed according to standard procedures at 25 ± 2°C, pH 7.0 ± 0.2 and a conductivity of 525 ± 50 mS (system water) with a 14:10 hour light-dark photoperiod in a ZebTec (Tecniplast, Buguggiate, Italy) recirculating zebrafish housing system. The system water is RO water amended with artificial sea salts (30g/ L Coral Ocean Plus salt from ATI GmBH, Germany and 30g/L NaHCO_3_ from Sigma-Aldrich, MO, US). The housing system from Tecniplast supplied the water and automatically adjusted the pH and conductivity. The fish were fed twice a day with SDS—Small Granular (Akronom, Budapest, Hungary) or Zebrafeed by Sparos (Olhão, Portugal).

Parents were spawned in pairs each week in double breeding tanks (Tecniplast, Buguggiate, Italy) that allow eggs to fall through the perforated bottom of the inner tank to avoid cannibalism by the parents. Ninety pairs provided embryos for these experiments. Parents were placed into the breeding tanks 15–16 hours prior to spawning, with males and females separated by a transparent removable wall. Immediately before spawning, the wall was removed, and the parents were allowed to spawn. Following spawning, the parents were removed from the breeding tanks.

All eggs fertilized from the breeding tanks were pooled, and then groups of 200 embryos were collected and incubated in 10-cm Petri-dishes at 26°C until treatment. The age of the embryos for the experiments was set to 26-somites or Prim-5 stage.

Each experimental embryo group was handled separately after the treatment until 5 day-post-fertilization (dpf) and the start of exogenous feeding. The water around the embryos was completely changed once a day. At the stage of 5 dpf, each group of larvae was individually placed into 15-cm Petri-dishes and fed once a day with a mixture of banana worms (*Panagrellus nepenthicola*) and SDS 100 feed dissolved in system water. Banana worms derived from the Banana Worm Starter Culture of the University of Veterinary Medicine, Budapest. The water around the larvae was completely changed once a day, 90 minutes after feeding. Larval development was monitored daily under a Leica M205FA stereomicroscope (Leica, Wetzlar, Germany) following the water change. Mortalities and possible developmental defects (tail malformations, pericardial edema, trunk curvature) were recorded. Health status was monitored daily, including normal feeding behavior and appetite, intact skin and normal social behavior. Groups of larvae were placed into 3.5-L culture tanks in the recirculating housing system on 15 dpf. The tanks were cleaned daily, and mortalities were recorded.

Studies were conducted in the approved laboratory animal unit of Department of Aquaculture of Szent István University, Hungary (permission number: PEI/001/1719-2/2015, issued by Government Office for Pest County, Hungary).

Experiments including the breeding procedure were approved by the Animal Welfare Committee of Szent István University, Hungary and by Government Office for Pest County, Hungary (permission number: XIV-1-001/2301-4/2012, issued to Szent István University, Hungary). We confirm that the embryo samples in this study did not originate from a third party.

All treatments in this study were conducted on embryos in accordance with the 3R principle while independently feeding larval forms and adult fish were reared based on the Directive 2010/63/EU of the European Parliament and of the Council. The health status was monitored daily. Regarding that survival rate was an endpoint as well, no euthanasia was applied during the experiments. At the end of the experiments a decision to keep a fish alive was taken in accordance with the Directive 2010/63/EU mentioned above. In case, selected fish would have been euthanized using an overdose of tricaine methane sulfonate (MS222) by prolonged immersion.

### PTAT treatment

**Fig 1:**
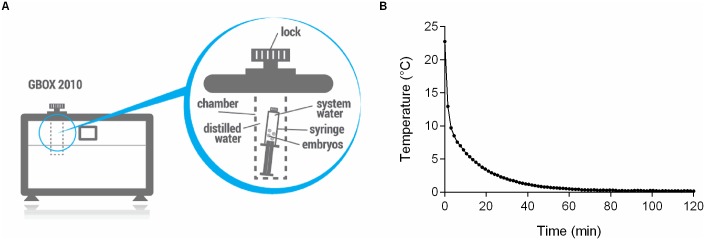
Schematic diagram of the hydrostatic pressure device and the chilling curve applied for zebrafish embryos. (A) The capacity of the pressure chamber is 100 cm^3^, the range of use is between 2 MPa and 90 MPa. The custom-made software runs the adjusted or cell-specific preconditioning treatment by controlling hydrostatic pressure build up, holding and coming back to atmospheric pressure and the temperature in the pressure chamber. The biological material is placed into an appropriate cell container (e.g. luer lock syringe) filled with the convenient fluid (this case by system water). After proper closing cell container is merged into the pressure filled with water. (B) Groups of 26-somites or Prim-5 stage embryos were placed on ice in zebrafish system water containing cryoprotectants. Cooling occurred according to the following cooling rates: 3.16°C/min for 4.5 minutes, 0.28°C/min for 22.5 minutes, 0.06°C/min for 30 minutes, and 0.01°C/min for 33 minutes. Embryos were kept at 0.0–0.3°C for additional 22.5 hours (24 hours in total).

Embryos were aspirated in 2-ml luer lock syringes (B.Braun Melsungen AB, Melsungen, Germany) in system water and locked by plastic luer lock caps. The syringes of the PTAT group were then placed into the pressure chamber of a computer-controlled, programmable hydrostatic pressure device (GBOX 2010, Applied Cell Technology Ltd., Budapest, Hungary, Fig 1A). The device was set to perform 5 MPa (one pascal (Pa), equal to one newton per square metre [N/m^2^ or kg·m^−1^·s^−2^] or 145,04·10^−6^ psi) hydrostatic pressure for 90 min, at 25°C. The pressure build-up in the chamber and depressurization to atmospheric pressure was set to 6 sec / 10 MPa. The settings of the pressure device have been optimized in the preparatory phase of this experiment. Several combinations of different pressure levels and durations have been tested and the optimal settings have been defined based on the dose-response assay (unpublished data).

### Chilled storage of embryos

Chilling was performed according to the modified protocol of Desai et al. [21]. Briefly, embryos were placed into 50-ml screw-cap centrifuge tubes (Axygen, Union City, CA, USA) containing 10 ml of chilling medium (system water supplied with 1 M methanol and 0.1 M sucrose as cryoprotectants) and placed on ice for 24 hours. The temperature was monitored (Fig 1B) and kept constant at 0.0–0.3°C during the entire chilling period. Cooling profile was measured using a K-type thermocouple connected to a Digi-Sense DualLogR thermometer (Eutech Instruments, Singapore). After the 24-hour exposure, embryos were transferred to a fine mesh dip net and washed with system water for approximately 30 sec. The embryos were then placed into 10-cm Petri-dishes, and further rearing was performed as described in section 2.1.

### Spawning

Animals were spawned separately in spawning tanks with Leopard danio (*Danio rerio var*. *frankei*) individuals at a sex ratio of 3:1 (three Leopard danio to one experimental fish) to keep track of the zebrafish during experiments. Spawned Leopard danio fish derived from the same source, from breeding unit of Department of Aquaculture, Szent István University, Gödöllő, Hungary. Following spawning, fish were removed from the spawning tanks, and embryos were incubated in 10-cm Petri-dishes until 10 dpf, as described in section 2.1.

### Experimental design

Fish embryos were pooled on each experimental day. Then, embryos were randomly divided and equally allocated to the experimental and control groups. Three consecutive experiments were planned.

#### Experiment I. Safety of the PTAT treatment

The aim of the experiment was to test whether PTAT treatment is safe and yields embryos that develop similarly to the untreated controls. Embryos were either treated with PTAT (PTAT group) or kept at atmospheric pressure (control group) for the same amount of time (n = 200 embryos per group) and then cultured for 30 days. Hatching and/or embryo survival was compared on 6, 10 and 30 dpf. The offspring morphology was compared on 10 and 30 dpf. Experiments were replicated four times.

#### Experiment II. Embryo survival and continued development after PTAT treatment and chilled storage

We hypothesized that PTAT treatment of embryos before chilled storage will increase their survival rate. Experimental groups (n = 200 per group) were PTAT preconditioned or incubated at 25°C, then placed at 0.0–0.3°C for 24 hours (PTAT chilled and control chilled groups, respectively) and then cultured for 30 days. A third, untreated unchilled group was used to monitor the breeding system.

Hatching rates on 6 dpf, survival rates every day, and morphology on 10 dpf and 30 dpf were evaluated. Experiments were replicated four times.

#### Experiment III. Embryo development to maturity and ability to produce healthy offspring after PTAT treatment and chilled storage

We hypothesized that PTAT treatment of embryos before chilled storage will actually enable them to develop to maturity and be able to produce healthy offspring. Four experimental groups were created. Chilled groups (n = 100 per experimental groups) were treated with PTAT or incubated at atmospheric pressure and then kept at 0.0–0.3°C for 24 hours (PTAT chilled group and control chilled group, respectively). Unchilled groups (n = 100 per group) were treated with PTAT or left at atmospheric pressure and then incubated at 25°C in parallel with the chilled storage of the chilled groups (PTAT group and control group, respectively). Subsequently, all groups were cultured separately until the proposed sexual maturity (~90–120 days), when the fish were spawned.

Ten fish from the unchilled groups and all fish from the PTAT chilled group that reached maturity (n = 5) were propagated with six mating attempts for six consecutive weeks. After fertilization, development of the offspring was evaluated on 5 dpf and 10 dpf. The morphological characteristics of day 10 larvae were recorded and quantified. Fertilization experiments were replicated six times.

The sample size used in each experiment was calculated based on the results of our pilot study which aimed to develop the most effective treatment protocol (two sample mean and standard deviation).

### Statistical analysis

All data were analyzed using GraphPad Prism 6 software (GraphPad Software, Inc., La Jolla, CA, USA). The survival data were analyzed by Kaplan-Meier nonparametric tests, nonparametric logrank tests and Cox-regression tests (using Stata/SE 14 software, StataCorp LP, College Station, TX, USA). To analyze morphology and fertility data, the appropriate ANOVA followed by Tukey’s or Sidak’s post-hoc test as required or Student’s paired t-test (when comparing two variables) were performed. A probability value (p) of less than 0.05 was considered significant. Values are presented as the mean ± SEM.

## Results

### Experiment I. Safety of PTAT treatment

The impact of PTAT treatment on embryo survival, development and morphology was studied by examining two matching groups of animals (control and PTAT) cultured according to standard housing conditions.

Embryo survival and further development was not adversely affected by PTAT. Control and PTAT-treated groups showed similar development without any differences in the hatching rate by 6 dpf (99.3 ± 0.3% for control and 98.7 ± 0.2% for PTAT). Similarly, survival was the same on 10 dpf (98.1 ± 0.6% for control and 95.4 ± 2.4% for PTAT) and 30 dpf (69.8 ± 0.8% for control and 69.1 ± 4.8% for PTAT) after fertilization. The morphological characteristics (prevalence of normal morphology, occurrence of tail abnormalities and pericardial edema) of the two groups were identical, both groups showed normal morphology.

### Experiment II. Embryo survival and development after PTAT treatment and chilled storage

The chilling sensitivity of 26-somites or Prim-5 stages zebrafish embryos with or without PTAT preconditioning was next examined. The effects of PTAT and chilling on development, hatching, survival and morphology were evaluated compared to control (chilled without PTAT preconditioning).

**Fig 2:**
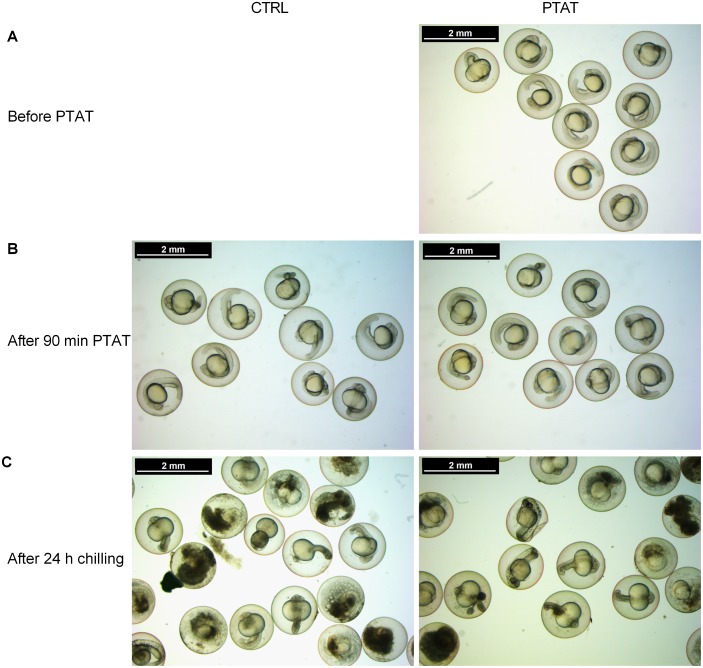
Appearance of zebrafish embryos before and after PTAT treatment and after chilling. Zebrafish embryos at 26-somites and Prim-5 stages were submitted to PTAT treatment (A). The preconditioning caused no morphological changes on embryos (B). The 24 hour long storage on ice arrested the development, after chilling (C) the embryos showed the same developmental stages as before. The beneficial effect of PTAT treatment was remarkable right away after the chilled storage.

Chilling for 24 hours has arrested the development of embryos and in both groups embryos remained in the 26-somites or Prim-5 embryo stages by the end of the chilling process (Fig 2).

**Fig 3:**
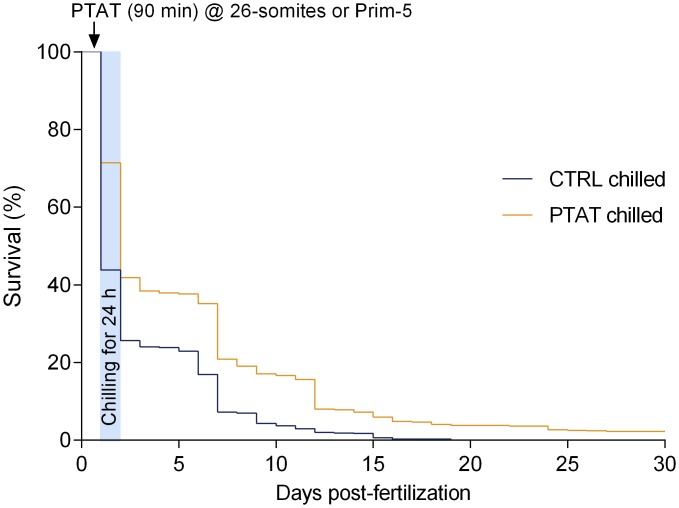
PTAT preconditioning decreases chilling sensitivity, and enhances embryo survival after chilling. Kaplan-Meier estimates of zebrafish embryo survival in the first 30 day-post-fertilization. 200–200 embryos were randomized to PTAT and control groups, experiments were replicated four times. Embryos at 26-somites and Prim-5 stages were exposed to PTAT for 90 minutes or incubated at atmospheric pressure, then were chilled on ice for 24h (PTAT chilled and CTRL chilled, respectively). No viable control embryo in the CTRL chilled group was found after 19 dpf in any of our experiments. PTAT preconditioning was statistically significant for fish survival (p<0.0001).

As it is shown in Fig 3, chilling survival was significantly improved by PTAT preconditioning. By 6 dpf, 37.6 ± 3.4% of embryos had hatched in the PTAT chilled group, compared to the 23.0 ± 3.8% for the control chilled group. By 10 dpf, heartbeat was detected in 17.1 ± 3.5% of PTAT chilled larvae compared to 4.3 ± 1.7% of controls. On one hand, all chilled controls died by 19 dpf. On the other hand, the average survival rate in the PTAT chilled group on 30 dpf was 2.3 ± 1.0%. Kaplan-Meier analysis and the logrank test based on daily individual data until 30 dpf (Fig 3) revealed that the PTAT preconditioning was statistically significant for fish survival (p<0.0001). A semi-parametric Cox-regression provided similar results (p<0.001).

**Fig 4:**
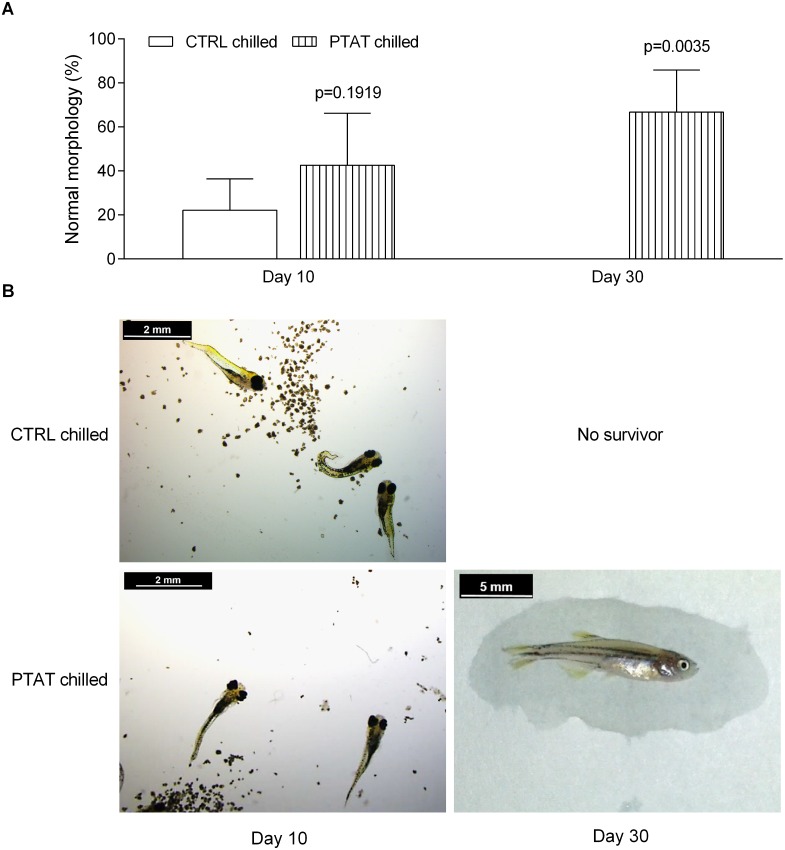
PTAT treatment improves the ratio of fish with normal morphology after 24 h chilled storage. Larval/fish morphology was evaluated on 10 dpf and 30 dpf. (A) Mean values (± SEM) of ratio of morphologically normal larvae/fish per experiments (four repetitions) are presented. Remarkably higher percentage of morphologically normal larvae/fish was detected in the PTAT-treated chilled group, compared to the untreated chilled controls. No viable fish was found in the control chilled groups on 30 dpf. (B) Illustration of morphological abnormalities detected on 10 and 30 dpf.

Embryo morphology was evaluated 10 days after fertilization by registering normal morphology, the occurrence of tail abnormalities and pericardial edema. More morphologically normal embryos were detected in the PTAT chilled group than in the control chilled group (42.5 ± 23.7% vs. 22.1 ± 14.3% normal morphology; p = 0.1919; Fig 4). By 30 dpf, 66.7 ± 19.2% of PTAT chilled individuals showed normal morphology.

### Experiment III. Embryo development to maturity and ability to produce healthy offspring after PTAT treatment and chilled storage

Knowing that PTAT preconditioning was successful, we next hypothesized that this treatment would enable fish to reach maturity and produce healthy offspring.

In the third experimental phase, all fish in the control chilled group died by 30 dpf (as in Exp. II), whereas the PTAT chilled group grew to maturity. Thus, fertility results, viability and morphology of the offspring were compared to the unchilled control and PTAT groups.

**Fig 5:**
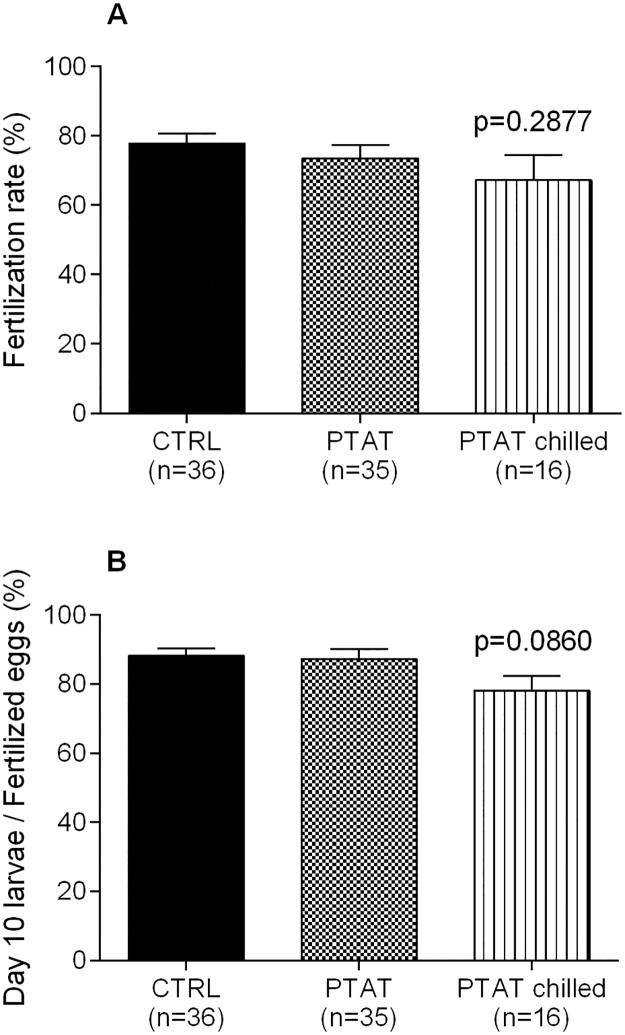
PTAT helps to preserve fertility potential of chilled embryos. (A) Average fertilizing capacity of adults developed from embryos chilled after PTAT (PTAT chilled), compared to the unchilled control (CTRL) and unchilled PTAT (PTAT) groups. The fertilization rates are comparable. (B) Percentage of viable offspring on 10 dpf. The offspring of fish derived from PTAT treated chilled embryos develop in the normal pace and have normal morphology. Mean values (± SEM) of four replicates are presented.

The two unchilled groups and the PTAT chilled group didn’t differ in terms of fertilization rates (77.8 ± 2.9% for control, 73.5 ± 4.0% for PTAT and 67.2 ± 7.3% for PTAT chilled group; Fig 5A), and the viability of offspring on 10 dpf (88.1 ± 2.2% for control, 87.3 ± 2.8% for PTAT, and 78.1 ± 4.3% for PTAT chilled group; Fig 5B). Larvae with normal morphology on 10 dpf were similar in all groups (data not shown).

## Discussion

The only promising way to preserve zebrafish embryos to date, despite all attempts, is the cryopreservation of isolated blastomeres. However, these cells do not develop after thawing, therefore germ-line chimerism was a suggested method to preserve embryonic genetic material [36].

As cryopreservation trials failed, most of the experiments focuses on developing cold- or chilled storage methods, however this area needs major developments as well. Lahnsteiner tested different temperature and exposure times at different stages of embryo development [16]. The maximal survival rate detected was from the Germ-ring stage to the Prim-25 stage after short (60–180 min) exposure at 4°C–8°C (above 70%). However, 5-somite stage embryos chilled at 1°C became nonviable within 6 hours.

A remarkable improvement was reported by Desai and his colleagues [37]. They tested the impact of chilling on 50% epiboly stage embryos exposed to 0°C for different times using methanol as a cryoprotectant. Survival was evaluated in terms of the hatching rate. As was shown, 3–6 hours of chilling resulted in no significant differences in hatching rates (over 85%), while 18 and 24 hours of exposure, even with cryoprotectant, significantly decreased the hatching rates (to less than 10%).

Compared to the former experiments a substantially different approach was taken in our present study. Instead of fine-tuning the various steps of the chilling/cryopreservation procedure, we applied a preconditioning treatment to the embryos before chilling. Thus, embryos gained increased tolerance to the effects of chilling, survived and developed significantly better compared to untreated controls.

In order to prove the beneficial effect of the PTAT preconditioning, we have tested the technology on a single selected developmental stage. Regarding that former results published by Liu et al. [38] and Lahnsteiner et al. [18] showed the highest chilling tolerance around 24 hour-post-fertilization stage, the chilling experiments were performed on embryos at this stage.

To prove the beneficial preconditioning effect, we conducted three experiments focusing on 1) the safety of PTAT treatment (Experiment I—no chilling was involved), 2) the efficiency of PTAT preconditioning to support embryo survival and continued development after chilled storage (Experiment II), and 3) the long-term effects from pretreatment reaching maturity as well as having offspring after the chilled storage (Experiment III).

Experiment I showed no difference in survival, development or morphology of the PTAT-treated zebrafish embryos compared to untreated controls. This study is in agreement with previous findings where gamete or embryo survival was not adversely affected by the optimal hydrostatic pressure treatment [reviewed in: 30,34,35].

As shown in Experiment II, PTAT preconditioning significantly improved chilling tolerance of 26-somites or Prim-5 stage zebrafish embryos. Our results demonstrate that embryos chilled without PTAT pretreatment are able to hatch; however, none of the untreated controls survived beyond 19 day-post-fertilization, while several in the PTAT-preconditioned group grew past 30 dpf. Moreover, the beneficial effects of the PTAT treatment were noticeable in the increased ratio of larvae with normal morphology.

In comparison, previous studies reported very limited chilling survival of embryos from zebrafish or other fish species even though they used either hatching [17,19–21] or stages close to hatching [39] as endpoints of the experiments. To our knowledge, the present study is the first to report that fish embryos survive until 30 dpf after 24 hours of chilled storage. These results indicated that PTAT preconditioning significantly improves fish embryo chilling and thus encouraged us to extend the examination period until maturity and to test spawning.

In the third experimental round (Experiment III), we investigated if PTAT-treated and chilled zebrafish embryos can develop to sexual maturity and produce viable offspring. Our findings suggest that not only does PTAT treatment improve chilling tolerance and enhance survival, but fish developed from PTAT-pretreated and chilled embryos preserve their fertility potential and are able to produce healthy offspring. Because offspring of chilled fish developed at a normal pace with normal morphology, PTAT is also a safe procedure regarding reproduction.

Currently, no direct information is available on the exact molecular mechanism of how PTAT preconditioning enhances the chilling tolerance of zebrafish embryos. However, the possible mechanism might be outlined based on scientific publications. As reported recently (23), zebrafish embryos possess the ability to improve their cold tolerance: mild cold stress preconditioning (16°C for 24 hours) of 96 hpf embryos could significantly increase the survival rate of forthcoming severe cold stress (exposure to 12°C). Gene ontology enrichment analysis revealed that RNA processing, ribosome biogenesis and protein catabolic processes were the most highly overrepresented pathways after cold-induction. Parallel to these results, hydrostatic pressure pretreatment (PTAT treatment) of bovine embryos increased the recovery rate of forthcoming vitrification accompanied by analogous gene expression changes: genes involved in RNA processing, cellular growth and proliferation were up-regulated, while genes belonging to cell death and apoptosis were down-regulated [26]. Increased gene expression of RNA processing and ribosome biogenesis processes indicates that embryos respond to the preconditioning and prepare cells for the upcoming detrimental procedures. Similarly, down-regulation of cell death or apoptotic processes, while up-regulating catabolic processes of denatured or misfolded proteins, can improve cellular integrity.

Unlike all other environmental stresses, the main advantage of hydrostatic pressure used in PTAT treatments is that it acts immediately and uniformly at each point of the sample. There are no penetration problems or gradient effects, and the PTAT can be applied with high precision, consistency and reliability [30].

Based on our results presented in this paper, we conclude that PTAT preconditioning has a significant beneficial effect to enhance general resistance and developmental competence of chilled zebrafish embryos. Regarding that the embryonic development is arrested during chilled storage this technology provides a solution to synchronize or to delay the development for experimental purposes. Moreover, treatment made it possible to create viable offspring, which is a unique and remarkable step forward. Further improvement of PTAT-chilling/cryopreservation procedures have the potential for application in zebrafish shipment and trade between laboratories as well as gene preservation.
